# Comparison of Polymer Clips Versus Endoloop Ligatures for Appendiceal Stump Closure in Laparoscopic Appendectomy

**DOI:** 10.7759/cureus.95024

**Published:** 2025-10-21

**Authors:** Madan Haravu Srikantegowda, Shilpashree Channasandra Shekar, Vinod Nayak S

**Affiliations:** 1 Department of General Surgery, Sri Chamundeshwari Medical College Hospital and Research Institute, Channapatna, IND

**Keywords:** appendectomy, appendicitis, endoloops, laparoscopy, polymeric clips

## Abstract

Objective: This study aimed to compare the safety and effectiveness of endoloop ligatures (ELs) (Ethicon India Pvt. Ltd., Mumbai, India) and polymer clips (PCs) (Medtronic India Pvt. Ltd., Hyderabad, India) for appendiceal stump closure during emergency laparoscopic appendectomy.

Methods: A prospective clinical study was conducted at Employees' State Insurance Corporation (ESIC) Medical College and Hospital, Hyderabad, India, including 70 patients who underwent laparoscopic appendectomy. Patients were randomly assigned to either the PC or EL group. Data collected included operative time, postoperative pain (measured using the visual analog scale), length of hospital stay, and postoperative complications.

Results: Baseline characteristics were comparable between the two groups. The PC group had a significantly shorter hospital stay (3.63 ± 0.77 vs. 4.20 ± 0.93 days; p = 0.007) and shorter operative time (61.46 ± 10.76 vs. 68.03 ± 8.80 minutes; p = 0.006) compared to the EL group. Postoperative pain scores were also significantly lower in the PC group at one, three, six, and 12 hours after surgery (p = 0.001). Although the overall incidence of complications did not differ significantly, abdominal pain and surgical site infections were less frequent in the PC group.

Conclusion: PCs are a safe and effective alternative to endoloops for appendiceal stump closure in laparoscopic appendectomy. They offer the advantages of reduced operative time, shorter hospital stays, and lower postoperative pain.

## Introduction

Laparoscopic surgery has transformed general surgical practice by offering several advantages over traditional open procedures, including reduced postoperative pain, shorter hospital stay, faster recovery, and improved cosmetic outcomes [1,2]. Among these advances, laparoscopic appendectomy has become the standard approach for managing acute appendicitis, one of the most common surgical emergencies worldwide. Secure closure of the appendiceal stump during laparoscopic appendectomy is critical, as inadequate sealing can result in severe postoperative complications such as peritonitis, intra-abdominal abscess, or stump leakage [3,4]. Several techniques are available for stump closure, including linear staplers, endoloop ligatures (ELs), and polymeric clips (PCs), such as Hem-o-lok clips (HCs). Although staplers are highly effective, their high cost often restricts their use, particularly in resource-limited settings.

ELs are widely employed because they are relatively easy to apply, cost-effective, and adaptable to different anatomical variations [5]. By applying consistent circumferential pressure, they may lower the risk of ischemic necrosis. ELs may offer better long-term biocompatibility because they leave no permanent foreign material in the body [5]. These features make endoloops particularly advantageous in low-resource environments and for less-experienced surgeons, where precision and affordability are essential.

Hem-o-lok polymer clips have emerged as an alternative due to their simplicity, safety, and efficiency. They enable rapid and secure stump closure, potentially reducing operative time, which is an essential consideration in emergency surgery [6,7]. Furthermore, HCs allow direct visual confirmation of placement, and their locking mechanism minimizes the risks of leakage and clip dislodgement, which are major postoperative concerns [8,9].

Although both techniques are widely used and considered safe, comparative studies have reported inconsistent findings [6-10]. A meta-analysis of randomized controlled trials found no statistically significant difference in overall postoperative complication rates; however, it noted a higher risk of intra-abdominal abscess with EL use (relative risk (RR) = 3.53) [10]. This suggests that while both methods are safe when performed correctly, subtle differences in outcomes may exist.

Given the extensive use of HCs and endoloops, particularly in emergency laparoscopic appendectomies, there remains a need for direct comparative evaluation to determine the optimal technique in terms of safety, cost-effectiveness, ease of application, and patient outcomes. Therefore, this study was designed to compare the effectiveness of ELs (Ethicon India Pvt. Ltd., Mumbai, India) and Hem-o-lok polymer clips (Medtronic India Pvt. Ltd., Hyderabad, India) for appendiceal stump closure during laparoscopic appendectomy to guide clinical decision-making and improve patient care.

## Materials and methods

Study design

This was a prospective observational study conducted in the Department of General Surgery at Employees' State Insurance Corporation (ESIC) Medical College and Hospital, Hyderabad, India, over a two-year period (September 2019 to September 2021). The objective was to compare outcomes in patients undergoing laparoscopic appendectomy, where the appendiceal stump was closed either with PCs or ELs, according to the surgeon’s preference.

Ethical clearance

Prior to study initiation, the protocol was submitted to the Institutional Ethics Committee (IEC) of ESIC Medical College and Hospital, Hyderabad, and ethical clearance was obtained. All participants were informed in detail about the study objectives, procedures, potential risks, and benefits. Written informed consent was obtained from each participant before inclusion, and participation was voluntary.

Eligibility criteria

Patients aged >18 years presenting with acute right iliac fossa pain and clinically or ultrasonographically confirmed acute or recurrent appendicitis were considered eligible. The Alvarado score [11] was used to stratify the probability of appendicitis and support clinical decision-making. Patients with systemic illnesses such as diabetes mellitus, hypertension, coronary artery disease (CAD), or thyroid disorders were also included, provided they met the other criteria.

Patients with a history of previous abdominal surgery, medical unfitness for pneumoperitoneum, pregnancy, neurological or pulmonary disorders, age <18 years, refusal to participate, or conversion to open appendectomy due to intraoperative complications were excluded.

Selection of study groups

Eligible patients undergoing laparoscopic appendectomy during the study period were prospectively observed. The method of appendiceal stump closure was determined by the operating surgeon according to routine practice and material availability. Patients were subsequently classified into two groups: those treated with PCs and those treated with ELs.

Sample size calculation

Sample size was calculated using the standard two-sample formula for comparing independent proportions:

$n_{\text{per group}}\;=\;\frac{\left(Z_{1-\alpha/2}\sqrt{2\bar{p}(1-\bar{p})}\;+\;Z_{1-\beta}\sqrt{p_1(1-p_1) + p_2(1-p_2)}\right)^2}{(p_1 - p_2)^2}$

where $p_1$ and $p_2$ are the expected proportions in the two groups, $\bar{p} = \frac{p_1 + p_2}{2}$, $Z_{1-\alpha/2}$ corresponds to a two-sided type I error $\alpha$, and $Z_{1-\beta}$ corresponds to the desired statistical power $1-\beta$.

For reproducibility, we report the assumptions used: two-sided $\alpha = 0.05$, power $= 80\%$, expected proportions $p_1 = 0.20$ (control group) and $p_2 = 0.50$ (intervention group). 

This required a sample size of 39 per group (total N ≈ 78).

During the study, eight participants withdrew consent or were lost to follow-up, resulting in a final sample of 70 patients (35 per group). Under the same assumptions, this provided an actual power of ~79%, only slightly below the target of 80%.

Method of data collection

All patients were assessed by a single trained investigator from the Department of General Surgery. Demographic data, clinical features, and laboratory results were collected from patient interviews and hospital records. Patients were evaluated in the emergency department with detailed history-taking, physical examination, Alvarado scoring, and investigations including complete blood count, urine analysis, and abdominal ultrasonography. A pre-anesthetic assessment was performed for all patients.

Intraoperative details recorded included operative time (incision to closure), intra-abdominal pressure gradient (documented in categories from 0 to 4 mmHg during stump closure), and intraoperative complications.

Postoperative outcomes were monitored. Pain was assessed using a visual analog scale (VAS) [12] at one, three, six, and 12 hours after surgery, where score 0 indicated no pain and score 10 indicated the worst pain. Abdominal pain was defined as localized or generalized discomfort persisting beyond the immediate postoperative period and requiring evaluation. Postoperative complications (e.g., abdominal pain, subcutaneous emphysema, surgical site infections) and duration of hospital stay were documented.

Statistical analyses

Data were entered into Microsoft Excel (Microsoft Corp., Redmond, USA) and analyzed using IBM SPSS Statistics v22 (IBM Corp., Armonk, USA). Descriptive statistics (means, standard deviations, and percentages) were calculated. For categorical variables (e.g., gender distribution, comorbidities, complications), the chi-square test or Fisher’s exact test was used as appropriate. For continuous variables (operative time, hospital stay, and VAS scores at different postoperative intervals), the independent Student’s t-test was applied. A p-value <0.05 was considered statistically significant.

## Results

The mean age was 29.37 ± 7.25 years in the PC group and 29.8 ± 6.53 years in the EL group. Male patients comprised 16 (45.7%) in the PC group and 19 (54.2%) in the EL group. The mean BMI was 23.2 ± 3.24 kg/m² in the PC group and 23.96 ± 2.21 kg/m² in the EL group. Diabetes mellitus was present in three (8.5%) versus four (11.4%) patients, hypertension in five (14.2%) versus six (17.1%) patients, and abnormal urine analysis in two (5.7%) patients in each group, with no significant differences between groups (all p > 0.05) (Table 1).

**Table 1:** Baseline characteristics of patients Values are presented as numbers (percentages) or mean ± standard deviation as appropriate. A p-value < 0.05 was considered statistically significant. t: independent t-test; c: chi-square test; f: Fisher's exact test; PC: polymer clip; EL: endoloop ligature

Parameter	Group		Test value	P-value
	PC (N = 35)	EL (N = 35)		
Age (years)	29.37 ± 7.25	29.8 ± 6.53	0.25^t^	0.8
Gender				
Male	16 (45.7%)	19 (54.2%)	0.23^c^	0.633
Female	19 (54.2%)	16 (45.7%)		
Body mass index (kg/m²)	23.2 ± 3.24	23.96 ± 2.21	1.11^t^	0.27
Diabetes mellitus				
No	32 (91.4%)	31 (88.5%)	0.46^c^	0.5
Yes	3 (8.5%)	4 (11.4%)		
Hypertension				
No	30 (85.7%)	29 (82.8%)	0.44^c^	0.5
Yes	5 (14.2%)	6 (17.1%)		
Abnormal urine analysis				
No	33 (94.2%)	33 (94.2%)	1^f^	1
Yes	2 (5.71%)	2 (5.71%)		

The mean pulse rate was 81.69 ± 5.16 bpm in the PC group and 80.57 ± 8.99 bpm in the EL group (t = 0.63, p = 0.53). The mean respiratory rate was 14.71 ± 1.53 cpm versus 14.83 ± 1.29 cpm (t = 0.34, p = 0.74). Mean systolic blood pressure was 125.26 ± 7.39 mmHg in the PC group and 124.91 ± 8.05 mmHg in the EL group (t = 0.19, p = 0.85), while mean diastolic blood pressure was 82.63 ± 3.32 mmHg and 82.51 ± 5.45 mmHg, respectively (t = 0.10, p = 0.92). The mean body temperature was 99.07 ± 1.28 °F in the PC group and 99.01 ± 0.90 °F in the EL group (t = 0.21, p = 0.83). Hemoglobin levels were 12.84 ± 1.40 g/dL and 12.95 ± 1.74 g/dL in the two groups, respectively (t = 0.26, p = 0.79). No significant differences were observed in these parameters (Table 2).

**Table 2:** Vital parameters of patients Values are presented as mean ± standard deviation. A p-value < 0.05 was considered statistically significant. PC: polymer clip; EL: endoloop ligature

Parameter	Group		Independent t-test value	P-value
	PC (N = 35)	EL (N = 35)		
Pulse rate (bpm)	81.69 ± 5.16	80.57 ± 8.99	0.63	0.53
Respiratory rate (cpm)	14.71 ± 1.53	14.83 ± 1.29	0.34	0.74
Systolic blood pressure (mmHg)	125.26 ± 7.39	124.91 ± 8.05	0.19	0.85
Diastolic blood pressure (mmHg)	82.63 ± 3.32	82.51 ± 5.45	0.10	0.92
Temperature (°F)	99.07 ± 1.28	99.01 ± 0.90	0.21	0.83
Hemoglobin (g/dL)	12.84 ± 1.40	12.95 ± 1.74	0.26	0.79

Acute appendicitis was present in 29 (82.8%) patients in both groups, while recurrent appendicitis occurred in six (17.1%) patients in each group (p = 1.00). The appendix was located in the pelvis in seven (20%) versus nine (25.7%) patients, post-ileal in three (8.5%) versus four (11.4%) patients, and retrocolic in 25 (71.4%) versus 22 (62.8%) patients in the PC and EL groups, respectively (p = 0.755) (Table 3).

**Table 3:** Comparison of type of appendicitis and appendix location between PC and EL groups Values are presented as numbers (percentages). A p-value < 0.05 was considered statistically significant. PC: polymer clip; EL: endoloop ligature

Parameter	Group		P-value
	PC (N = 35)	EL (N = 35)	
Appendicitis type			
Acute	29 (82.8%)	29 (82.8%)	1
Recurrent	6 (17.1%)	6 (17.1%)	
Appendix location			
Pelvic	7 (20%)	9 (25.7%)	0.755
Post-ileal	3 (8.5%)	4 (11.4%)	
Retrocolic	25 (71.4%)	22 (62.8%)	

A pressure gradient of 0 mmHg was observed in 13 (37.1%) patients in the PC group and none in the EL group. A gradient of 1 mmHg was noted in 18 (51.4%) patients in the PC group versus seven (20%) in the EL group, while a 2 mmHg gradient was observed in four (11.4%) versus 13 (37.1%) patients, respectively. Higher gradients were seen only in the EL group, with 10 (28.5%) patients showing 3 mmHg and five (14.2%) patients showing 4 mmHg (Table 4).

**Table 4:** Comparison of pressure gradient between PC and EL groups Values are presented as numbers (percentages). PC: polymer clip; EL: endoloop ligature

Parameter	Group		Total (N = 70)
	PC (N = 35)	EL (N = 35)	
Pressure gradient (mmHg)			
0	13 (37.1%)	0 (0%)	13 (18.5%)
1	18 (51.4%)	7 (20%)	25 (35.7%)
2	4 (11.4%)	13 (37.1%)	17 (48.5%)
3	0 (0%)	10 (28.5%)	10 (14.2%)
4	0 (0%)	5 (14.2%)	5 (7.1%)

Among postoperative complications, subcutaneous emphysema occurred in one (2.8%) patient in the PC group and three (8.5%) patients in the EL group (p = 0.614). Abdominal pain occurred in three (8.5%) patients with PCs and five (14.5%) with ELs (p = 0.710). Surgical site infection was reported in one (2.8%) patient in the PC group compared to three (8.5%) patients in the EL group (p = 0.614) (Table 5).

**Table 5:** Comparison of postoperative complications between PC and EL groups Values are presented as numbers (percentages). A p-value < 0.05 was considered statistically significant. f: Fisher's exact test; c: chi-square test; PC: polymer clip; EL: endoloop ligature

Parameter	Group		Test value	P-value
	PC (N = 35)	EL (N = 35)		
Subcutaneous emphysema				
No	34 (97.1%)	32 (91.4%)	0.26^f^	0.614
Yes	1 (2.8%)	3 (8.5%)		
Abdominal pain				
No	32 (91.4%)	30 (85.7%)	0.14^c^	0.71
Yes	3 (8.5%)	5 (14.5%)		
Surgical site infection				
No	34 (97.1%)	32 (91.4%)	0.26^f^	0.614
Yes	1 (2.8%)	3 (8.5%)		

The mean duration of hospital stay was significantly shorter in the PC group (3.63 ± 0.77 days) compared to the EL group (4.20 ± 0.93 days; t = -2.80, p = 0.007). Operative time was also significantly less with PCs (61.46 ± 10.76 vs. 68.03 ± 8.80 minutes; t = -2.87, p = 0.006). Postoperative pain scores (VAS) were consistently lower in the PC group at one hour (1.46 ± 0.51 vs. 2.46 ± 0.51; t = -8.24, p = 0.001), three hours (1.63 ± 0.49 vs. 3.00 ± 0.003; t = -15.0, p = 0.001), six hours (2.25 ± 0.57 vs. 4.74 ± 0.44; t = -20.0, p = 0.001), and 12 hours (4.09 ± 0.70 vs. 6.20 ± 0.41; t = -14.7, p = 0.001) (Table 6).

**Table 6:** Clinical outcomes and VAS scores between PC and EL groups Values are presented as mean ± standard deviation. A p-value < 0.05 was considered statistically significant. * indicates statistical significance. VAS: visual analog scale; PC: polymer clip; EL: endoloop ligature

Parameter	Group		Independent t-test value	P-value
	PC (N = 35)	EL (N = 35)		
Duration of hospital stay (days)	3.63 ± 0.77	4.2 ± 0.933	-2.80	0.007*
Operative time (minutes)	61.46 ± 10.76	68.03 ± 8.8	-2.87	0.006*
VAS score at one hour	1.46 ± 0.505	2.46 ± 0.505	-8.24	0.001*
VAS score at three hours	1.63 ± 0.49	3 ± 0.003	-15.0	0.001*
VAS score at six hours	2.25 ± 0.565	4.74 ± 0.443	-20.0	0.001*
VAS score at 12 hours	4.09 ± 0.702	6.2 ± 0.406	-14.7	0.001*

## Discussion

Laparoscopic appendectomy is the standard surgical approach for managing appendicitis, the most common acute abdominal condition requiring surgery [1]. Several techniques have been described for appendiceal stump closure, with endoloops traditionally being the most frequently used [3]. Recently, PCs have attracted attention as a cost-effective and time-saving alternative [4]. This study aimed to evaluate the safety and effectiveness of ELs versus PCs for appendiceal stump closure during emergency laparoscopic appendectomy.

The results of this study are consistent with several earlier investigations comparing ELs and PCs. The significantly shorter operative time in the PC group (61.46 ± 10.76 minutes) compared with the EL group (68.03 ± 8.80 minutes, p = 0.006) aligns with prior research. For example, in a retrospective analysis, Lee et al. [13] reported that HCs significantly reduced operative time (64.5 vs. 71.5 minutes, p = 0.027). Similarly, Wilson et al. [14] found that PCs facilitated faster closure times. Kumar et al. [15] also demonstrated a 10-minute reduction in operative time with PCs compared to endoloops in a pediatric cohort of 277 patients (p < 0.001). The meta-analysis further confirmed this time-saving effect, reporting a statistically significant advantage in surgical duration in favor of PCs (standardized mean difference (SMD) 0.37; p < 0.00001) [15].

In our study, patients in the PC group had a significantly shorter hospital stay (3.63 ± 0.77 days) than those in the EL group (4.20 ± 0.93 days, p = 0.007). These findings are consistent with Ihnát et al. [16], who reported fewer hospital days in the PC group. However, some meta-analyses have found no significant difference in hospital stay between the two methods, suggesting that outcomes may vary depending on patient characteristics or institutional policies [15].

A particularly notable finding in our study was the consistently lower postoperative pain scores in the PC group across all measured intervals (one, three, six, and 12 hours), with p-values <0.001 throughout. This observation aligns with Badgurjar et al. [17], who reported that patients treated with HCs experienced less severe pain and faster recovery. The reduced pain may be attributable to shorter closure time and less tissue manipulation required with PCs. Our study is relatively distinctive, as previous investigations have directly assessed VAS scores at multiple time points.

Although postoperative complications such as subcutaneous emphysema, abdominal pain, and surgical site infections were not statistically different between groups, a trend toward fewer complications was observed in the PC group. Hue et al. [18] reported higher intra-abdominal abscess and surgical site infection rates in patients with ELs. In fact, endoloops have been associated with an increased risk of intra-abdominal abscess formation (RR = 3.53) [10]. Nevertheless, a meta-analysis of randomized controlled trials found no significant difference in overall postoperative complication rates, supporting the general safety of both techniques when properly applied [19].

Another noteworthy finding in our study was the difference in pressure gradient patterns. Higher intra-abdominal pressures were observed more frequently in the EL group, which may suggest a more secure seal or reduced leakage with PCs. While few studies have directly examined this parameter, our results highlight a potentially novel area of investigation and contribute to the broader understanding of the physiological implications of different closure techniques.

Finally, evidence also supports the economic benefits of PCs. Akbiyik et al. [20] and Badgurjar et al. [17] reported that PCs are more cost-effective and efficient than ELs, especially in resource-limited settings. These findings reinforce the clinical advantages identified in our study, showing that PCs offer both procedural and recovery-related benefits without increasing costs or complication rates.

Limitations

This study has several limitations that should be acknowledged. First, the sample size was relatively small (70 patients), which may limit the generalizability of the findings to broader and more diverse populations. Second, as the trial was conducted at a single center, the results may be influenced by institutional practices and may not fully capture variations in surgical expertise or resource availability across different settings. Third, the follow-up was limited to the immediate postoperative phase, and long-term outcomes such as late abscess formation or adhesive bowel obstruction were not evaluated. Additionally, all procedures were performed by a single trained investigator, which ensured consistency but may reduce external validity, as outcomes could differ with surgeons of varying experience. Finally, a detailed cost analysis was not undertaken, although PCs are generally considered more economical in resource-limited environments.

## Conclusions

In conclusion, this study demonstrates that PCs are a safe and effective alternative to ELs for appendiceal stump closure during laparoscopic appendectomy. Both groups were comparable in baseline demographic and clinical characteristics, minimizing potential confounding effects. However, the PC group consistently showed superior outcomes, including significantly shorter operative time, reduced hospital stay, and lower postoperative pain scores at all measured intervals. Although complication rates did not differ significantly, a trend toward fewer adverse events was observed with PCs. These findings suggest that PCs offer surgical efficiency, faster recovery, and improved patient comfort, making them a valuable option for appendiceal stump closure in routine practice and resource-limited settings.
